# Giant Borderline Phyllodes Tumor of the Breast: A Case Report

**DOI:** 10.7759/cureus.60251

**Published:** 2024-05-14

**Authors:** George Mpourazanis, Haralambos V Harissis, Konstantinos Seretis, Petros Papalexis, Ioannis Korkontzelos, Romanos Vogiatzis, Ioannis Kosmas, Anastasia Zagaliki, Panagiotis Tsirkas

**Affiliations:** 1 Department of Obstetrics and Gynecology, Ioannina State General Hospital “G. Chatzikosta”, Ioannina, GRC; 2 Department of Surgery, University Hospital of Ioannina, Ioannina, GRC; 3 Department of Plastic Surgery, University Hospital of Ioannina, Ioannina, GRC; 4 Unit of Endocrinology, First Department of Internal Medicine, Laiko General Hospital, National and Kapodistrian University of Athens, Athens, GRC; 5 Department of Medicine, Faculty of Health Sciences, Aristotle University of Thessaloniki, Thessaloniki, GRC; 6 Department of Dermatology, Ernst-Moritz-Arndt University of Greifswald, Greifswald, DEU

**Keywords:** giant phyllodes tumor, giant borderline phyllodes tumor, breast reconstruction, borderline phyllodes tumor, phyllodes tumor

## Abstract

Borderline phyllodes tumor is a rare and benign form of breast cancer with malignant potential. According to the World Health Organization (WHO), phyllodes tumor is classified into three categories: benign, borderline, and malignant. The treatment of phyllodes tumor is wide focal excision combined with radiotherapy and chemotherapy in certain cases. Herein, we report a 47-year-old female who presented with a giant borderline mass approximately 19.5 x 16.9 x 9.3 cm in size. From medical history, we noticed that the mass begun to develop during puberty. Wide focal excision of the tumor and immediate implant-based reconstruction with free nipple graft was performed, with the tumor specimen measuring 16.5 x 14.2 x 8.7 cm. Histological examination reported a borderline phyllodes tumor, and in this case, the patient did not undergo adjuvant treatment.

## Introduction

According to scientific literature, phyllodes tumors are rare fibroepithelial breast neoplasms presenting with an incidence rate lower than 1% of breast tumors and all tumors [1]. They originate from the connective stromal tissues of the breast and show a prominent intracanalicular architectural pattern, characterized by a leaf-like appearance covered by double-layer epithelial elements [2]. Phyllodes tumor size varies and could reach up to 60 cm in diameter. Of these tumors, 20% have a diameter greater than 10 cm and are considered giant phyllodes tumors [3,4]. Most often, they present between the ages of 40 and 50, but in rare cases, they could present during puberty [5]. Their recurrence rate is 5-15% [6].

The World Health Organization (WHO) graded diagnostic criteria about phyllodes tumors, which are classified as benign, borderline, and malignant based on stromal hypercellularity, nuclear atypia, stromal overgrowth, stromal mitotic activity, tumor margins, and malignant heterogeneous components [7]. The National Comprehensive Cancer Network (NCCN) states that there is no stipulation concerning the criteria for classifying these subtypes [8]. The exact etiology of phyllodes tumors remains unknown. Between epithelial and stromal, there are biological markers that overexpress estrogen receptors, p53, c-kit, Ki-67, endothelin-1, epidermal growth factor receptor, and heparan sulfate. The Wnt-β-catenin pathway has a leading role in the growth of the normal gland and tumorigenesis of the mammary tissue. In addition, there is a theory that claims the evolution that the stroma develops a self-determining growth and outgrowth of any epithelial effect, leading to malignancy. Furthermore, contemporary theories mention that there are genetic imbalances within the tumor consistent with intratumoral heterogeneity, and subclones within histologically benign phyllodes tumors that reoccur or metastasize. Therefore, this tumor may have unpredictable clinical behavior. It is mentioned that it has a familiar association and females with germline Tp53 mutation pose an increased risk for developing phyllodes tumors [9-11]. Moreover, it is associated with syndromes, such as the Li-Fraumeni syndrome [12]. 

We report the case of a 47-year-old woman presenting with a left breast giant borderline phyllodes tumor. The phyllodes tumor existed from puberty until the age of 47 without any sign of metastasis confirming the nature and behavior of those tumors. That is why this case is worth describing.

## Case presentation

**Figure 1:**
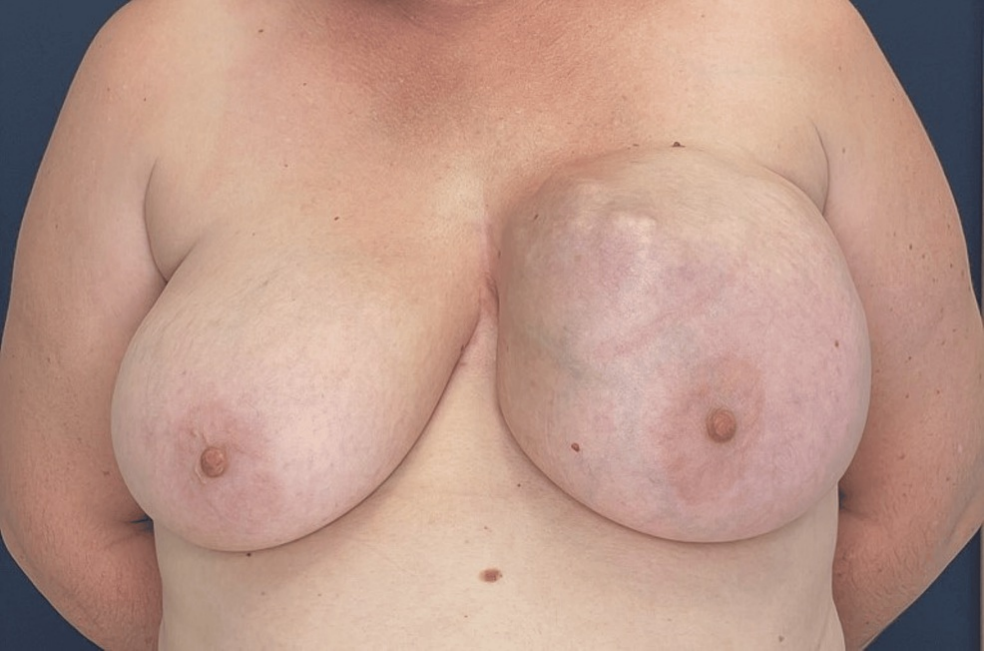
Preoperative image of the patient's breast

A 47-year-old Caucasian woman presented to the outpatient clinic of the breast surgery unit complaining of a mass in the left breast that had gradually increased in size since puberty and causing discomfort (Figure 1). Her medical history included two vaginal deliveries. She had never had a smear test. She had never received hormonal therapy. Her BMI was 28 kg/m^2^. The patient had no smoking or alcohol habits and did not mention any medication taken. On physical examination, a large mass measuring 19.5 x 16.9 x 9.3 cm was found at the left breast. 

**Figure 2:**
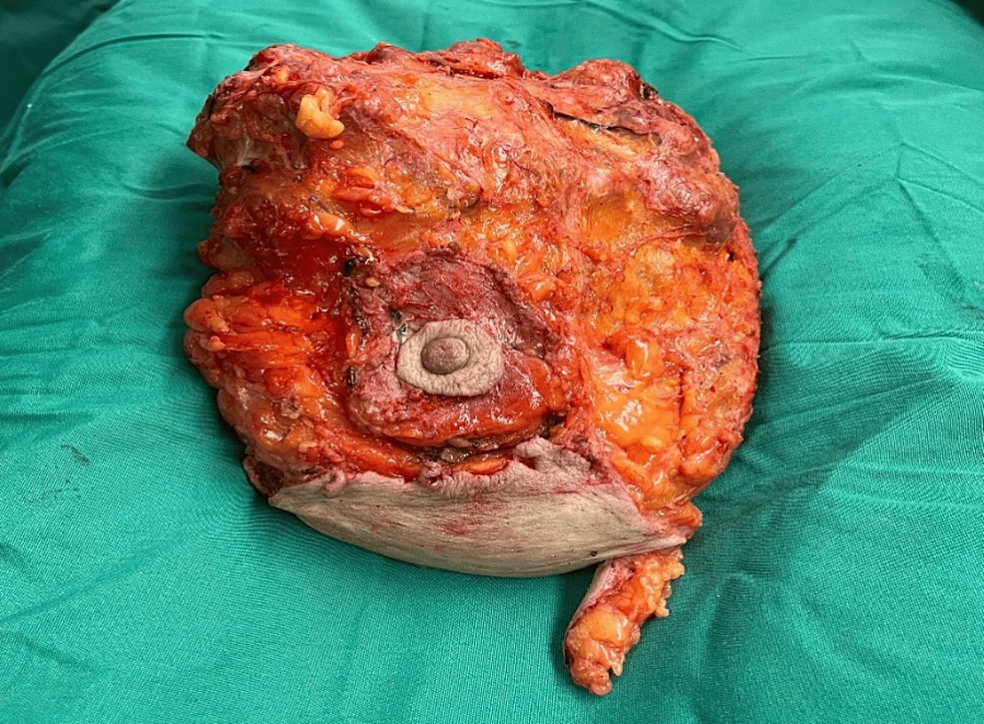
Macroscopic appearance of the excised tumor (dimensions: 19.5 cm x 17 cm x 9.3 cm)

At the theatre, the patient received general anesthesia and was arranged in a supine position. Total mastectomy of the left breast with immediate implant-based breast reconstruction was performed using a dermal sling to cover the lower part of the implant, while the nipple-areola complex (NAC) was removed along with the specimen, dissected, and used directly as a graft to the reconstructed breast. The upper, inner, lateral, and posterior surgical excision margins were <0.1 cm. Immediate implant-based reconstruction with free nipple graft was also performed. The NAC was removed en bloc with the specimen due to the size of the tumor, the breast ptosis, and the thin skin remaining above the tumor, which was inadequate to be used as a pedicle for the NAC. The NAC was then repositioned as a free nipple graft, directly above the reconstructed breast, to the pectoralis muscle and dermal sling, covering the implant. The specimen measured 19.5 x 17 x 9.3 cm (Figure 2), and histological sections showed growth of a fibroepithelial breast neoplasm with phyllodes configuration to the largest extent.

**Figure 3:**
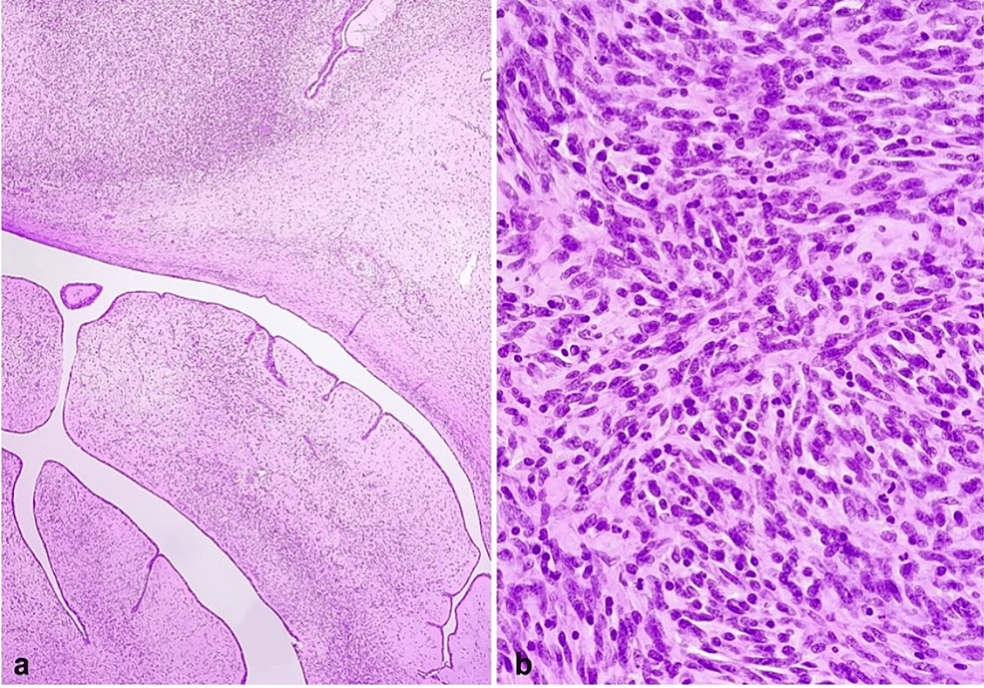
A: Fibroepithelial neoplasm, at low-power view, with leaf-like fronds capped by luminal epithelial and myoepithelial cell layers, accompanied by sites of stromal hypercellularity (H&E, 40x). B: Higher magnification of cellular stromal component with mild to moderate atypia and mitotic figures (H&E, 400x).

**Figure 4:**
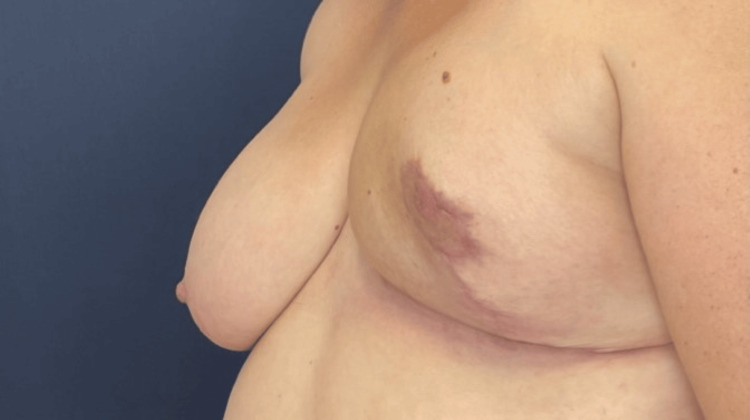
Postoperative image of the patient's breast

The laboratory results are listed in Table 1. Microscopic examination revealed a fibroepithelial neoplasm with a phyllodes configuration and spindle-cell stroma exhibiting variable cellularity (Figure 3a, 3b). Stromal cellular atypia ranged from mild to focally severe and mitotic activity was high, with over 10 mitotic counts per 10 high-power fields in several areas. Stromal overgrowth was identified in a restricted area of one histological slide. Tumor borders were primarily well-defined, while incipient permeation was considered in a single site. No malignant heterologous elements were recognized. A final diagnosis of a borderline phyllodes tumor was made. The postoperative image of the breast is shown in Figure 4.

**Table 1:** Laboratory test before and after the operation WBC: white blood cell; HGB: hemoglobin; HCT: hematocrit; INR: international normalized ratio; PLT: platelet; CRP: C-reactive protein; AST: aspartate transferase; ALT: alanine transaminase; ALP: alkaline phosphatase; AMY: amylase; LDH: lactate dehydrogenase; CA 125: cancer antigen 125; CA 15-3: cancer antigen 15-3; CA 19-9: cancer antigen 19-9; CEA: carcinoembryonic antigen

Parameter	Day 0 (admission and operation)	Day 1	Day 4 (exit day)	Follow-up (12 months)	Reference number
WBC	8.98 k/μL	14 k/μL	10 k/μL	9 k/μL	4-11 k/μL
Neutrophils	77.9%	90%	60%	50%	40-75%
HGB	13.9 g/dl	10.5 g/dl	11.8 g/dl	14.7 g/dl	11.8-17.8 g/dl
HCT	43%	35%	38%	45%	36-52%
INR	1.00	1.00	-	-	0.8-1.2
PLT	305 k/μl	240 k/μl	259 k/μl	334 k/μl	140-450 k/μl
CRP	2 mg/dl	1.5 mg/dl	0.5 mg/dl	0.2 mg/dl	0-0.80 mg/dl
AST	32 IU/l	29 IU/l	24 IU/l	22 IU/l	10-35 IU/l
ALT	27 IU/l	30 IU/l	19 IU/l	18 IU/l	10-35 IU/l
ALP	50 IU/l	70 IU/l	45 IU/l	45 IU/l	30-125 IU/l
AMY	37 IU/l	45 IU/l	36.8 IU/l	36 IU/l	0-90 IU/l
LDH	280 U/L	350 U/L	240 U/L	200 U/L	115-230 U/L
CA 125	25.9 U/mL	-	-	12.7 U/mL	<35 U/mL
CA 15-3	20.8 U/mL	-	-	14.3 U/mL	<31.3 U/mL
CA 19-9	24.4 U/mL	-	-	10 U/mL	<37 U/mL
CEA	2 ng/mL	-	-	1 ng/mL	<5 ng/gL

## Discussion

A phyllodes tumor is a rare breast tumor presenting as a mobile, uniteral, single, rounded painless, and swelling mass. Interestingly, the left breast is usually more affected than the right one [13], and it could grow into a huge mass within a span of weeks or years [14]. These tumors may be malignant causing metastatic carcinomas in the lungs, skeleton, heart, and liver, but the possibility of metastasis is very rare [15]. The prognosis of phyllodes tumors can be further improved since local recurrence usually occurs within the first years following surgery, especially in cases of incomplete surgical excision [16].

Histologically, the features of this type of phyllodes tumor may include well-defined tumor borders, moderate stromal cellularity, mild or moderate stromal atypia, mitotic activity of 5-9 counts per 10 HPFs, and focal stromal overgrowth, whereas malignant heterologous elements should be absent [17]. It was aforementioned that, in our patient, cellular damage varied with a sufficient number of mitoses while no malicious heterologous elements were recognized.

According to a meta-analysis of 807 patients with borderline phyllodes tumors, 782 (96.9%) underwent surgery and the recurrence rate was 16.7%. Meta-regression analysis showed no significant difference in the recurrence rate of borderline phyllodes tumors between patients receiving postoperative adjuvant radiotherapy compared to those not receiving radiotherapy (P = 0.626). The study also noted that surgical margins of 1 mm revealed from analysis (odds ratio (OR): 0.4, 95% confidence interval (CI): 0.27-0.61) and 1 cm (OR: 0.45, 95% CI: 0.15-0.85) resulted in significantly higher recurrence rates [18].

A retrospective cohort study showed that the recurrence rates from patients with borderline phyllodes tumors with a surgical margin of ≥1 mm and <1 mm showed a significantly higher recurrence rate (OR: 0.40, 95% CI: 0.27-0.61) in the <1-mm than in the ≥1-mm margin group [19]. The ideal method for treating giant borderline phyllodes tumors is wide focal surgical excision with a margin of 1 cm of the breast tissue [17]. In our case, the surgical margins were <1 cm of the breast tissue. Furthermore, long-term follow-up is of great importance since inadequate excision of the tumor may lead to recurrence [20].

## Conclusions

We reported a rare giant borderline phyllodes tumor of the breast. The exact transition mechanism from benign to malignant is still unclear and remains to be further studied in the future. Prompt intervention/treatment and close follow-up are crucial to therapeutically reduce the likelihood of recurrence and metastasis.
